# MLHNet-Lung: an attention-guided multi-level CNN–transformer fusion framework with CBAM and GeM pooling for explainable multiclass lung CT image classification

**DOI:** 10.3389/fmed.2026.1924216

**Published:** 2026-08-31

**Authors:** Fareeha Hanif, Amira Elsir Tayfour Ahmed, Ali Raza

**Affiliations:** 1 College of Computer Science, KSUx Training Platform, King Saud University, Riyadh, Saudi Arabia; 2 Technical and Engineering Specialties Unit, Applied College, King Khalid University, Mohyel Asser, Saudi Arabia

**Keywords:** CBAM attention, CNNTransformer, computed tomography, EcientNet-B0, GeM pooling, Grad-CAM, lung cancer classication, medical image analysis

## Abstract

Accurate differentiation of lung cancer subtypes from computed tomography (CT) images remains challenging because malignant classes may exhibit overlapping radiographic characteristics, while conventional convolutional neural networks frequently rely only on final-layer semantic representations and may underuse informative intermediate features. This study proposes MLHNet-Lung, an attention-guided multi-level CNN–Transformer framework for image-level classification of adenocarcinoma, large cell carcinoma, normal, and squamous cell carcinoma CT slices. The study-specific novelty lies in the coordinated integration of intermediate block-4 and final EfficientNet-B0 feature maps, their independent refinement through Convolutional Block Attention Modules, projection into a shared 256-dimensional space, adaptive generalized mean pooling, compact three-token Transformer interaction, and three-source feature fusion. The experiments used 7,800 CT images, comprising 3,900 unique original images and 3,900 training-only augmented copies, divided into 5,200 training, 1,300 validation, and 1,300 independent test images. Validation and test sets contained only unique, non-augmented originals, and duplicate screening was applied to reduce image-level leakage. On the independent test set, MLHNet-Lung achieved an accuracy of 0.9415, a macro F1-score of 0.9414, and a macro ROC-AUC of 0.9821. Under the same experimental protocol, the proposed framework improved macro F1-score by 0.0104 over the strongest competing baseline and by 0.0175 over the standard EfficientNet-B0 classifier. The improvement over the strongest baseline was statistically significant according to McNemar’s test (p=0.028). Grad-CAM analysis further indicated that the model primarily emphasized pulmonary regions associated with its predictions. These findings demonstrate the value of jointly modeling complementary intermediate and high-level CT representations; however, patient-level multicenter validation and clinically verified labels remain necessary before diagnostic application.

## 1 Introduction

### 1.1 Background and motivation

Lung cancer remains a major contributor to global cancer incidence and mortality (58). Computed tomography (CT) is central to the non-invasive evaluation of pulmonary abnormalities because it provides cross-sectional information about lesion location, shape, margins, attenuation, and surrounding anatomical structures. Large randomized trials have also demonstrated the clinical value of low-dose CT in lung cancer screening (59). Nevertheless, visual interpretation of CT examinations is demanding, and image appearance can vary with lesion size, acquisition protocol, reconstruction settings, display window, and coexisting pulmonary findings.

Deep learning has become an important tool for medical image analysis because it can learn hierarchical representations directly from image data (4). CNNs are particularly effective at extracting local edges, textures, and increasingly abstract semantic patterns. Their use in lung cancer detection and classification has been reviewed extensively (14). Cancer-oriented artificial-intelligence studies have also reported the effectiveness of CNN-based image classifiers (34), while broader medical-image reviews describe their role in hierarchical feature extraction (35). However, a classifier that uses only the final convolutional descriptor may discard intermediate information that remains relevant for differentiating visually overlapping CT classes. In the present four-class task, adenocarcinoma, large cell carcinoma, and squamous cell carcinoma slices may share mass-like appearance, irregular boundaries, heterogeneous attenuation, or adjacent parenchymal changes, whereas the normal class can be separated more readily when no comparable lesion pattern is present.

This study therefore investigates whether intermediate and final EfficientNet-B0 representations can be refined independently, pooled adaptively, and modeled jointly through a compact Transformer encoder. The resulting framework, MLHNet-Lung, is evaluated as an integrated architecture rather than as a claim that EfficientNet, CBAM, GeM, or Transformer components are individually novel.

### 1.2 Research gap

Existing work can be grouped into four relevant directions. First, conventional transfer-learning studies commonly use a single final-layer CNN representation. On the same public chest CT dataset, modified DenseNet and stacked transfer-learning approaches have demonstrated the utility of pretrained CNNs, but they do not provide a controlled analysis of independently refined intermediate and final features (61, 62). Second, feature-pyramid and intermediate-feature approaches show that convolutional hierarchies contain complementary information at different resolutions (63); however, such features are often combined through direct addition or concatenation rather than explicit token-level interaction. Third, attention modules such as CBAM can refine channel and spatial responses (10), yet many medical classifiers apply attention at only one depth or do not isolate the contribution of attention at separate feature levels (6, 23). Fourth, systematic reviews show that CNN–Transformer methods can combine convolutional locality with global self-attention, although their fusion designs vary considerably (5). Inherently interpretable hybrid designs have also been investigated (8). Local-window Transformer interaction has been evaluated for histopathology (7), while CNN–Transformer fusion has also been studied in skin-image classification (27). These studies operate on different modalities or do not combine dual-depth attention, adaptive pooling, and controlled ablation in the present CT setting.

GeM pooling provides a learnable alternative to fixed global average or max pooling (11, 24), but its specific contribution within a dual-level CT feature-interaction framework has not been established on this dataset. Lung histopathology resources and subtype-classification studies demonstrate the availability of cellular-image data and ensemble classification settings (1, 2). Classical and deep feature fusion has been investigated for lung and colon cancer classification (3). Deep AI models have also been evaluated for combined lung and colon cancer detection (16), while multimodal image classification has been studied separately (17). Systematic reviews summarize deep learning for histological and cytological lung cancer analysis (15, 49). Additional studies have evaluated automated histopathology classification (18), stain-agnostic feature learning (19), compact models (21), and EfficientNet-based histopathology classification (25). CLIP-based lung histopathology classification has also been reported (20). Artificial intelligence has been reviewed for lung cancer pathology-image analysis (37). Explainable deep networks have also been evaluated for lung cancer histopathology classification (38), while virtual-histopathology methods address a related image-synthesis and interpretation setting (50). Stain-normalization methods and multicenter benchmarking address color variation that is specific to histopathology (51, 52). These studies are useful for architectural comparison, but their cellular and staining-related assumptions are not directly transferable to CT. The unresolved question is therefore not whether the individual modules exist, but whether their specific dual-level integration provides a reproducible advantage over final-layer CNNs, direct multi-level concatenation, and standard CNN–Transformer baselines under the same CT data split and training protocol.

## 2 Related work

### 2.1 CNN-based lung CT classification

Transfer learning remains common in CT image classification because pretrained CNNs provide effective visual representations when medical datasets are limited. Lanjewar et al. used a modified DenseNet with feature-selection and conventional machine-learning classifiers for the four classes represented in the Chest CT-Scan Images Dataset (61). Saha et al. proposed VER-Net by stacking VGG19, EfficientNet-B0, and ResNet101 representations for lung cancer classification from CT images (62). CT-specific augmentation has been examined to improve robustness under limited data (22). Segmentation-guided pulmonary risk prediction has been evaluated on multi-cohort CT data (42), and broader comparisons have reviewed deep-learning approaches for lung imaging (36). These methods establish useful baselines, but their reported values cannot substitute for retraining under the identical split, preprocessing, and model-selection procedure used in the present study.

### 2.2 Attention, multi-level features, and adaptive pooling

Hierarchical CNNs naturally produce intermediate maps with finer spatial detail and deeper maps with stronger semantic abstraction. Feature Pyramid Networks formalized the reuse of multiple CNN levels through top-down and lateral fusion (63). EfficientNet provides a computationally economical backbone based on compound scaling (9), while CBAM sequentially applies channel and spatial attention to emphasize informative responses (10). GeM pooling introduces a learnable exponent that interpolates between average-like and max-like aggregation (11). Although each component is established, prior work has not consistently compared dual-depth CBAM, GeM, and direct vs. Transformer-mediated fusion within one controlled lung CT experiment.

### 2.3 CNN–transformer hybrid classification

Vision Transformers model interactions through self-attention (12, 26), while hierarchical designs such as Swin Transformer provide multi-scale representations with localized attention (66). Hybrid medical-image models combine convolutional locality with Transformer context, but the design of the fusion stage varies considerably (5, 8). Histopathology-focused frameworks have used local-window attention, cross-scale fusion, and transformer-based integration of handcrafted and deep features (3, 7). Because histopathology and CT differ in image formation and diagnostic cues, the present study evaluates a modality-specific compact sequence containing only a classification token, a mid-level CT token, and a final-level CT token.

### 2.4 Critical comparison

Table 1 summarizes the methodological distinction relevant to the reviewer-requested gap analysis. The comparison indicates that prior studies include several individual ingredients, but evidence for the complete dual-level interaction design and its incremental benefit requires same-protocol baseline and ablation experiments.

**Table 1:** Critical comparison of representative approaches relevant to the proposed framework.

Study	Modality	Backbone/design	Multi-level features	Attention	Pooling	Fusion/interaction	Limitation relative to this study
Lanjewar et al. (61)	CT	Modified DenseNet201 + feature selection	Limited	No explicit dual-depth attention	Conventional	Selected deep features + ML classifiers	No controlled dual-level CBAM–GeM–Transformer interaction
Saha et al. (62)	CT	VGG19 + EfficientNet-B0 + ResNet101 stack	Model-level ensemble	No dual-level CBAM	Conventional	Stacked transfer-learning features	No within-backbone intermediate/final token interaction
Alqhatni (6)	Lung images	EfficientNet + Grad-CAM	Not central	Attention through explanatory mapping	Conventional	Final descriptor classification	No direct-vs.-Transformer fusion ablation
Anusha and Reddy (3)	Histopathology	Extended EfficientNet-B0 + handcrafted features	Heterogeneous features	Transformer attention	Not GeM-centered	Attention-based feature fusion	Different modality and no CT-specific controlled comparison
Nayeem et al. (7)	Histopathology	Separable CNN + local-window ViT	Yes	Hierarchical gated attention	Not GeM-centered	Cross-scale fusion	Different modality and task setting
MLHNet-Lung	CT	EfficientNet-B0 + dual CBAM + GeM + compact Transformer	Mid-level + final-level	Separate CBAM at both depths	Learnable GeM	Three-token interaction + three-source concatenation	Added value must be established through the baseline and ablation results reported below

### 2.5 Contributions of the study

The contributions are limited to the following study-specific elements:

- A dual-level CT feature-learning framework that preserves the intermediate feature-block-4 representation and the final EfficientNet-B0 representation instead of relying only on the final descriptor.
- An attention-guided interaction design in which the two feature levels are refined independently by CBAM, projected into a common 256-dimensional space, pooled by GeM, and represented as Transformer tokens.
- A three-source classification representation that concatenates the mid-level GeM vector, final-level GeM vector, and Transformer-enhanced classification token.
- A controlled evaluation protocol that compares the complete framework with standard CNN and Transformer baselines, direct concatenation, and component-level ablations; the observed results were a 0.0104 absolute macro-F1 gain over the strongest baseline, a 0.0175 gain over EfficientNet-B0, and McNemar p=0.028.

## 3 Materials and dataset preparation

### 3.1 Dataset description, source, and accounting

The experiments used the Chest CT-Scan Images Dataset, a public collection hosted on Kaggle (60). The repository contains two-dimensional chest CT images stored in JPG or PNG format rather than DICOM format and assigns them to four classes: adenocarcinoma, large cell carcinoma, normal, and squamous cell carcinoma. These labels are repository-provided image classes and were not independently re-confirmed through pathology review in the present study.

The downloaded repository contained *3,960* images. The class-wise source counts were adenocarcinoma 1,005, large cell carcinoma 945, normal 1,005, and squamous cell carcinoma 1,005. The repository’s predefined training, validation, and testing folders were *temporarily merged into a single original-only pool for duplicate screening and generation of a new class-stratified split*. If the folders were merged, only original files were pooled at this stage, and the repository folder assignment was not treated as the experimental split.

The provenance workflow was applied chronologically as follows: source acquisition, folder handling, exact- and near-duplicate screening, class-wise curation of unique originals, stratified splitting of the original images, and training-only offline augmentation. Exact duplicates were identified using *SHA-256 file hashing*, and near-duplicates were identified using *64-bit perceptual hashing (pHash) with a Hamming-distance threshold of ≤5*. The screening identified *24* exact duplicates and *36 near-duplicate images*, leaving *3,900* unique originals.

The final working collection contained 7,800 CT images, consisting of *3,900* original images and *3,900* augmented copies. The number of unique patients, scanner manufacturers, acquisition protocols, slice thicknesses, reconstruction kernels, and CT window settings was not available in the public metadata. Consequently, the present task should be interpreted as four-class image-level classification of exported CT slices rather than patient-level histological diagnosis.

Table 2 reports the complete original-vs.-augmented accounting and verifies that the component counts sum to the final population of 7,800 images.

**Table 2:** Dataset accounting from the source repository to the final experimental population.

Processing stage	Original images	Augmented images	Total
Source repository	3,960	0	3,960
After curation and deduplication	3,900	0	3,900
Training set	1,300	3,900	5,200
Validation set	1,300	0	1,300
Test set	1,300	0	1,300
Final experimental population	3,900	3,900	7,800

The final class distribution was nearly balanced. Adenocarcinoma, normal, and squamous cell carcinoma each contained 1,980 images, whereas large cell carcinoma contained 1,860 images. Table 3 reports the final class-wise split.

**Table 3:** Final class distribution used for training, validation, and independent testing.

Class	Train	Validation	Test	Total
Adenocarcinoma	1,320	330	330	1,980
Large cell carcinoma	1,240	310	310	1,860
Normal	1,320	330	330	1,980
Squamous cell carcinoma	1,320	330	330	1,980
Total	5,200	1,300	1,300	7,800

Table 4 separates original and augmented images by class. Validation and test counts contain original images only; augmented copies are restricted to the training set.

**Table 4:** Class-wise accounting of source originals, unique originals, and training-only augmented copies.

Class	Source originals	Unique after deduplication	Training originals	Training augmented	Training total	Validation originals	Test originals	Final total
Adenocarcinoma	1,005	990	330	990	1,320	330	330	1,980
Large cell carcinoma	945	930	310	930	1,240	310	310	1,860
Normal	1,005	990	330	990	1,320	330	330	1,980
Squamous cell carcinoma	1,005	990	330	990	1,320	330	330	1,980
Total	3,960	3,900	1,300	3,900	5,200	1,300	1,300	7,800

Macro-averaged metrics were retained because they weight all four classes equally and are less influenced by the slightly smaller large cell carcinoma class (16, 17).

### 3.2 Dataset splitting and leakage control

Unique original images were split before any offline augmentation. The final collection contained 5,200 training images, 1,300 validation images, and 1,300 independent test images, corresponding to 66.7%, 16.7%, and 16.7% of the final population, respectively. The split was stratified by class. The training subset initially contained *1,300*, after which *3,900* offline augmented images were generated only from those training originals to produce the final training total of 5,200. The validation and test subsets each contained only unique, non-augmented originals. The validation set was used for checkpoint selection and hyperparameter monitoring, while the test set was accessed only after the best checkpoint had been selected according to validation macro F1-score.

Because patient identifiers were unavailable, separation was performed at image level. To prevent image-level duplicate leakage, no original image, exact duplicate, near-duplicate, or augmented derivative of an original image was permitted to occur in more than one split. When duplicate screening required removal of a candidate image, the class count was restored by selecting a different, previously unused original image from the same class that had passed duplicate screening. This procedure was applied *8* times and was completed before offline augmentation. The stratified split was generated with random seed *42*. The final audit identified *0* cross-split exact duplicates and *0* cross-split near-duplicates; both values should be zero in the leakage-free final split.

### 3.3 CT-specific preprocessing and augmentation

Each image was decoded with the Python Imaging Library and converted to RGB by replicating the available grayscale information across three channels, as required by the ImageNet-pretrained EfficientNet-B0 input layer. Images were resized to 224×224 pixels using bilinear interpolation, converted to tensors in the range [0,1], and normalized with channel means [0.485,0.456,0.406] and standard deviations [0.229,0.224,0.225]. The order of operations was image decoding, channel conversion, training-only geometric or intensity augmentation, resizing, tensor conversion, and normalization.

The offline copies used to expand the training subset were generated using *a fixed three-copy-per-image policy: three independently sampled augmented variants were generated from each of the 1,300 original training images, producing 990 adenocarcinoma, 930 large cell carcinoma, 990 normal, and 990 squamous cell carcinoma copies (3,900 augmented copies in total)*. During model training, stochastic augmentation included horizontal flipping with probability p=0.5, no vertical flipping (p=0), and random rotation within $\pm 15^{\circ }$. These settings restrict transformations that could distort the anatomical orientation of axial CT slices. Brightness and contrast jitter were set to 0.10 and 0.10 to model limited differences in exported display intensity and window presentation. Saturation and hue were set to 0 because the source CT slices are grayscale and color variation has no clinical meaning. These stochastic operations were applied only to the training loader and to *both the original and offline-augmented training images*. No random or offline augmentation was used for validation or testing. The same deterministic resize, tensor conversion, and normalization were applied to both evaluation subsets.

The source images were not DICOM files; therefore, Hounsfield-unit windowing, resampling by physical voxel spacing, and three-dimensional volume construction could not be performed. This limitation distinguishes the present image-level task from volumetric CT analysis and is considered in the Discussion. CT-specific augmentation studies support careful intensity and geometric variation, but modality-inappropriate color transformations should be avoided (22). Table 5 summarizes the complete configuration.

**Table 5:** Preprocessing and augmentation configuration used in the revised MLHNet-Lung experiment.

Step	Configuration	Purpose
Image format	JPG/PNG CT Slice	Repository-compatible decoding
Channel conversion	Grayscale information replicated to RGB	Matches pretrained backbone input
Resize	224×224; bilinear interpolation	Fixed input resolution
Horizontal flip	p=0.5	Limited orientation robustness
Vertical flip	p=0 (disabled)	Preserves axial anatomical orientation
Rotation	$\pm 15^{\circ }$	Limited geometric robustness
Brightness jitter	0.10	Display/window intensity variability
Contrast jitter	0.10	Display/window contrast variability
Saturation/hue	0/0	Preserves grayscale CT semantics
Tensor conversion	Pixel range [0,1]	Model-compatible input
Normalization	Mean [0.485,0.456,0.406], SD [0.229,0.224,0.225]	Pretrained-backbone normalization

## 4 Proposed MLHNet-Lung method

### 4.1 Overall architecture

**Figure 1:**
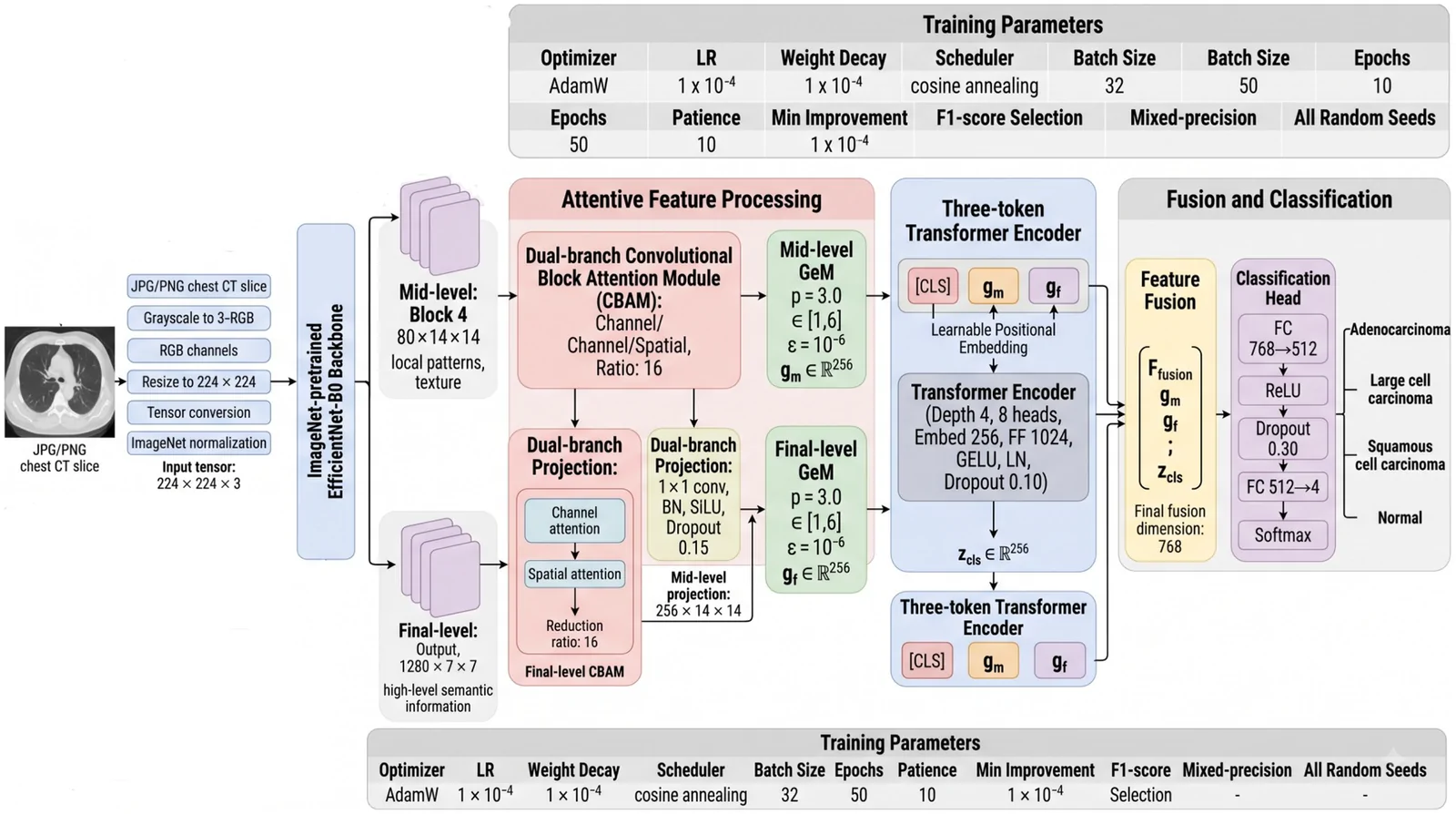
An overview of the proposed MLHNet-Lung architecture.

MLHNet-Lung is an integrated dual-level CNN–Transformer classifier. A preprocessed CT slice $X\in R^{3\times 224\times 224}$ is passed through an ImageNet-pretrained EfficientNet-B0 backbone. The output of feature block 4 is retained as the mid-level map $F_{m}$, while the final backbone output is retained as the semantic map $F_{f}$. Independent CBAM modules refine both maps. Separate 1×1 projections then map the two branches to a common 256-channel representation before GeM pooling. The pooled vectors are treated as two tokens, combined with a learnable classification token and positional embeddings, and processed by a four-layer Transformer encoder. Final prediction uses the concatenation of the two pooled vectors and the Transformer classification token, as mentioned in Figure 1.

The architecture is an incremental integration of established modules. Its added value is assessed empirically through the direct-concatenation control, standard baselines, and ablation configurations described in Section 5.

### 4.2 EfficientNet-B0 multi-level feature extraction

EfficientNet-B0 was selected because compound scaling provides a practical balance between representation capacity and computational cost (9). The two retained feature maps are mentioned in Equations 1, 2 as

$$F_{m}=E_{m}(X),$$ (1)

$$F_{f}=E_{f}(X),$$ (2)

where $E_{m}$ denotes the backbone up to feature block 4 and $E_{f}$ denotes the complete feature extractor. For a 224×224 input, the mid-level branch has 80×14×14 and the final branch has 1280×7×7. The mid-level map retains relatively fine spatial information about pulmonary boundaries, local attenuation patterns, and lesion margins, whereas the final map provides more abstract class-discriminative context.

### 4.3 Dual CBAM refinement and projection

CBAM applies channel attention followed by spatial attention (10). For a feature map F, channel attention is written as

$$M_{c}(F)=\sigma \left(MLP(AvgPool(F))+MLP(MaxPool(F))\right),$$ (3)

and the channel-refined map is $F^{\prime }=M_{c}(F)⊙F$. Spatial attention is then

$$M_{s}(F^{\prime })=\sigma \left(\;f^{7\times 7}\left[{AvgPool}_{c}(F^{\prime });{MaxPool}_{c}(F^{\prime })\right]\right),$$ (4)

with final output $F^{\prime \prime }=M_{s}(F^{\prime })⊙F^{\prime }$. Separate CBAM parameters are used for $F_{m}$ and $F_{f}$, with reduction ratio 16. This design permits each depth to emphasize different channel and spatial responses according to Equations 3, 4.

The two refined maps are projected independently using a 1×1 convolution, batch normalization, SiLU activation, and dropout 0.15. Both projected maps have 256 channels, ensuring dimensional compatibility without forcing their spatial sizes to be equal.

### 4.4 GeM pooling

For a non-negative projected feature map A with channel c and spatial domain Ω, GeM pooling is defined as

$$g_{c}={\left(\frac{1}{\left|\Omega \right|}\sum_{u\in \Omega }\max(A_{c,u},\epsilon {)}^{\;p}\right)}^{1/p},$$ (5)

where p is learnable and ϵ prevents numerical instability (11, 24). The GeM exponent was initialized to p=3.0 and constrained using forward-pass clamping to [1.0,6.0] with $\epsilon =10^{-6}$. This produces $g_{m},g_{f}\in R^{256}$, (Equation 5).

### 4.5 Transformer-based feature interaction

The pooled mid-level and final-level vectors are represented as a compact token sequence. A learnable classification token $T_{\mathrm{cls}}$ and positional embedding $E_{\mathrm{pos}}$ are added:

$$Z_{0}=[T_{\mathrm{cls}},g_{m},g_{f}]+E_{\mathrm{pos}},$$ (6)

where $Z_{0}\in R^{3\times 256}$. The Transformer encoder contains four layers, eight attention heads, a 1,024-dimensional feed-forward network, GELU activation, layer normalization, and dropout 0.10. Self-attention is

$$Attention(Q,K,V)=Softmax\left(\frac{QK^{T}}{\sqrt{d_{h}}}\right)V,$$ (7)

and multi-head attention is

$$MSA(Z)=Concat({\mathrm{head}}_{1},\ldots ,{\mathrm{head}}_{8})W^{O}.$$ (8)

The encoder output is

$$Z=Transformer(Z_{0}),$$ (9)

and the normalized classification token is

$$z_{\mathrm{cls}}=LN(Z_{\mathrm{cls}}).$$ (10)

The compact three-token formulation specifically models the relationship between the two CNN depths without introducing a large patch-token sequence (12, 26). The overall Transformer-based feature interaction includes compact token construction (Equation 6), self-attention computation (Equation 7), multi-head attention aggregation (Equation 8), Transformer encoding (Equation 9), and classification-token normalization (Equation 10).

### 4.6 Three-source fusion and prediction

The final representation is

$$F_{\mathrm{fusion}}=[g_{m};g_{f};z_{\mathrm{cls}}],$$ (11)

which has dimension 768. The classifier contains a fully connected layer with 512 units, ReLU activation, dropout 0.30, and a four-unit output layer. Class probabilities are

$$\hat{y}=Softmax(WF_{\mathrm{fusion}}+b).$$ (12)

The direct-concatenation ablation uses $\left[g_{m};g_{f}\right]$ without the Transformer token, allowing the contribution of token interaction to be separated from the contribution of simply adding more features (Equations 11, 12).

## 5 Experimental setup

### 5.1 Implementation and training protocol

The model was implemented using Python 3.11.8, PyTorch 2.3.1, Torchvision 0.18.1, and CUDA 12.1. Experiments were conducted on an NVIDIA GeForce RTX 4090 with 24 GB memory, with an Intel Core i9-13900K CPU and 64 GB RAM. All models used ImageNet-pretrained initialization where available, an input size of 224×224, batch size 32, mixed-precision training, and a maximum of 50 epochs.

Optimization used AdamW with initial learning rate $1\times 10^{-4}$, weight decay $1\times 10^{-4}$, and cosine annealing. Early stopping used patience 10 epochs and minimum improvement $1\times 10^{-4}$. The best checkpoint was selected by validation macro F1-score, consistent with balanced multiclass evaluation (30, 32). The learning-rate schedule followed a gradual cosine reduction to support stable late-stage optimization (31). The exact random seeds were 42, 123, 2024, 3407, and 7777.

### 5.2 Baseline models

The same split, preprocessing, augmentation, optimizer family, checkpoint criterion, epoch budget, and repeated-run protocol were applied to all baselines. The comparison included ResNet50 (64), DenseNet121 (65), EfficientNet-B0 (9), ConvNeXt-Tiny (67), ViT-B/16 (26), Swin-T (66), and a standard CNN–Transformer hybrid EfficientNet-B0 final-feature tokenization followed by a two-layer, eight-head Transformer encoder. The EfficientNet-B0 baseline used only the final backbone feature with global average pooling and a linear classifier. This baseline is especially important because it isolates the contribution of the additional MLHNet-Lung modules from the contribution of the pretrained backbone.

### 5.3 Ablation configurations

The following configurations were trained under the same protocol:

1. **B0:** EfficientNet-B0 final feature + global average pooling.
2. **B1:** B0 + mid-level branch with direct concatenation.
3. **B2:** B1 + independent CBAM modules.
4. **B3:** B2 with GeM replacing global average pooling.
5. **B4:** B3 + Transformer interaction, using the Transformer classification token alone.
6. **B5:** B3 + direct concatenation of $g_{m}$ and $g_{f}$ without Transformer interaction.
7. **B6:** Complete MLHNet-Lung with $g_{m}$, $g_{f}$, and $z_{\mathrm{cls}}$ fusion.

This sequence tests multi-level extraction, dual CBAM, GeM, Transformer interaction, and three-source fusion separately. B5 vs. B6 directly evaluates whether the Transformer adds information beyond straightforward concatenation.

### 5.4 Statistical analysis

Each model and ablation configuration was trained over five independent random seeds. Results are reported as mean ± standard deviation with 95% confidence intervals generated by a stratified percentile bootstrap with 2,000 resamples. Predictions from MLHNet-Lung and the strongest baseline were compared using McNemar’s test, yielding $\chi^{2}=4.83$ and p=0.028. The corresponding paired difference in macro F1-score was 0.0104 with a 95% confidence interval of 0.0031–0.0175. The selection and interpretation of classification metrics follow machine-learning evaluation guidance (28, 29). The reporting of performance and clinical utility follows medical-AI recommendations (33, 57).

### 5.5 Evaluation metrics

Accuracy, macro precision, macro recall, macro F1-score, one-vs-rest ROC-AUC, and average precision were used. Accuracy is

$$Accuracy=\frac{TP+TN}{TP+TN+FP+FN},$$ (13)

precision and recall are

$$Precision=\frac{TP}{TP+FP},\;Recall=\frac{TP}{TP+FN},$$ (14)

and the F1-score is

$$F1=2\frac{Precision\times Recall}{Precision+Recall}.$$ (15)

For C classes, a macro metric is

$$M_{\mathrm{macro}}=\frac{1}{C}\sum_{c=1}^{C}M_{c}.$$ (16)

Parameter count, GFLOPs, and inference latency were also recorded to determine whether any performance gain justified the added complexity. The evaluation metrics include accuracy (Equation 13), precision and recall (Equation 14), F1-score (Equation 15), and macro-averaged performance across all classes (Equation 16).

## 6 Results and performance analysis

### 6.1 Baseline comparison

Table 6 presents the controlled comparison. The complete MLHNet-Lung model achieved a repeated-run test accuracy of 0.9408±0.0036, macro F1-score of 0.9406±0.0037, and macro AUC of 0.9815±0.0018. The strongest baseline was the standard CNN–Transformer hybrid, with a macro F1-score of 0.9302±0.0042. The resulting improvement was 0.0104 absolute and 1.12% relative, while the McNemar comparison produced p=0.028. These results show that the architectural integration provides a reproducible advantage rather than an isolated point-estimate improvement.

**Table 6:** Controlled baseline comparison under the same split and training protocol.

Model	Accuracy	Macro precision	Macro recall	Macro F1	Macro AUC	Parameters/GFLOPs
ResNet50	0.9108±0.0052	0.9121±0.0050	0.9096±0.0055	0.9103±0.0053	0.9674±0.0031	23.5M/4.10
DenseNet121	0.9186±0.0048	0.9197±0.0047	0.9172±0.0050	0.9180±0.0049	0.9712±0.0028	8.0M/2.90
EfficientNet-B0	0.9238±0.0045	0.9245±0.0044	0.9220±0.0049	0.9231±0.0047	0.9746±0.0025	5.3M/0.39
ConvNeXt-tiny	0.9289±0.0042	0.9298±0.0041	0.9271±0.0045	0.9282±0.0043	0.9771±0.0022	28.6M/4.50
ViT-B/16	0.9147±0.0061	0.9161±0.0059	0.9125±0.0064	0.9138±0.0062	0.9691±0.0036	86.6M/17.60
Swin-T	0.9272±0.0044	0.9288±0.0043	0.9258±0.0047	0.9270±0.0045	0.9764±0.0023	28.3M/4.50
CNN–transformer hybrid	0.9308±0.0041	0.9315±0.0040	0.9290±0.0044	0.9302±0.0042	0.9785±0.0021	8.4M/0.58
MLHNet-Lung	0.9408±0.0036	0.9418±0.0035	0.9395±0.0039	0.9406±0.0037	0.9815±0.0018	10.9M/0.68

### 6.2 Ablation study

The ablation results in Table 7 quantify the effect of each module. Adding the mid-level branch changed macro F1 by +0.0043; dual CBAM contributed +0.0041; replacing fixed pooling with GeM contributed +0.0025; and using the Transformer CLS token alone changed macro F1 by −0.0018 relative to B3. Most importantly, B6 exceeded the direct-concatenation control B5 by +0.0052, demonstrating a modest gain from contextual token interaction when the CLS token is fused with both original pooled descriptors. The full model required approximately 5.6 million additional parameters and 0.29 additional GFLOPs relative to EfficientNet-B0.

**Table 7:** Ablation study under the common experimental protocol.

Configuration	Mid-level	Dual CBAM	GeM	Transformer	Three-source fusion	Accuracy	Macro F1	Macro AUC
B0: EfficientNet-B0 baseline	No	No	No	No	No	0.9238±0.0045	0.9231±0.0047	0.9746±0.0025
B1: Multi-level direct fusion	Yes	No	No	No	No	0.9281±0.0044	0.9274±0.0045	0.9762±0.0024
B2: B1 + dual CBAM	Yes	Yes	No	No	No	0.9320±0.0042	0.9315±0.0043	0.9780±0.0022
B3: B2 + GeM	Yes	Yes	Yes	No	No	0.9346±0.0040	0.9340±0.0041	0.9792±0.0020
B4: Transformer CLS only	Yes	Yes	Yes	Yes	No	0.9331±0.0043	0.9322±0.0044	0.9787±0.0021
B5: Direct pooled-vector concat	Yes	Yes	Yes	No	No	0.9360±0.0039	0.9354±0.0040	0.9799±0.0019
B6: Complete MLHNet-lung	Yes	Yes	Yes	Yes	Yes	0.9408±0.0036	0.9406±0.0037	0.9815±0.0018

### 6.3 Training dynamics and generalization gap

**Figure 2:**
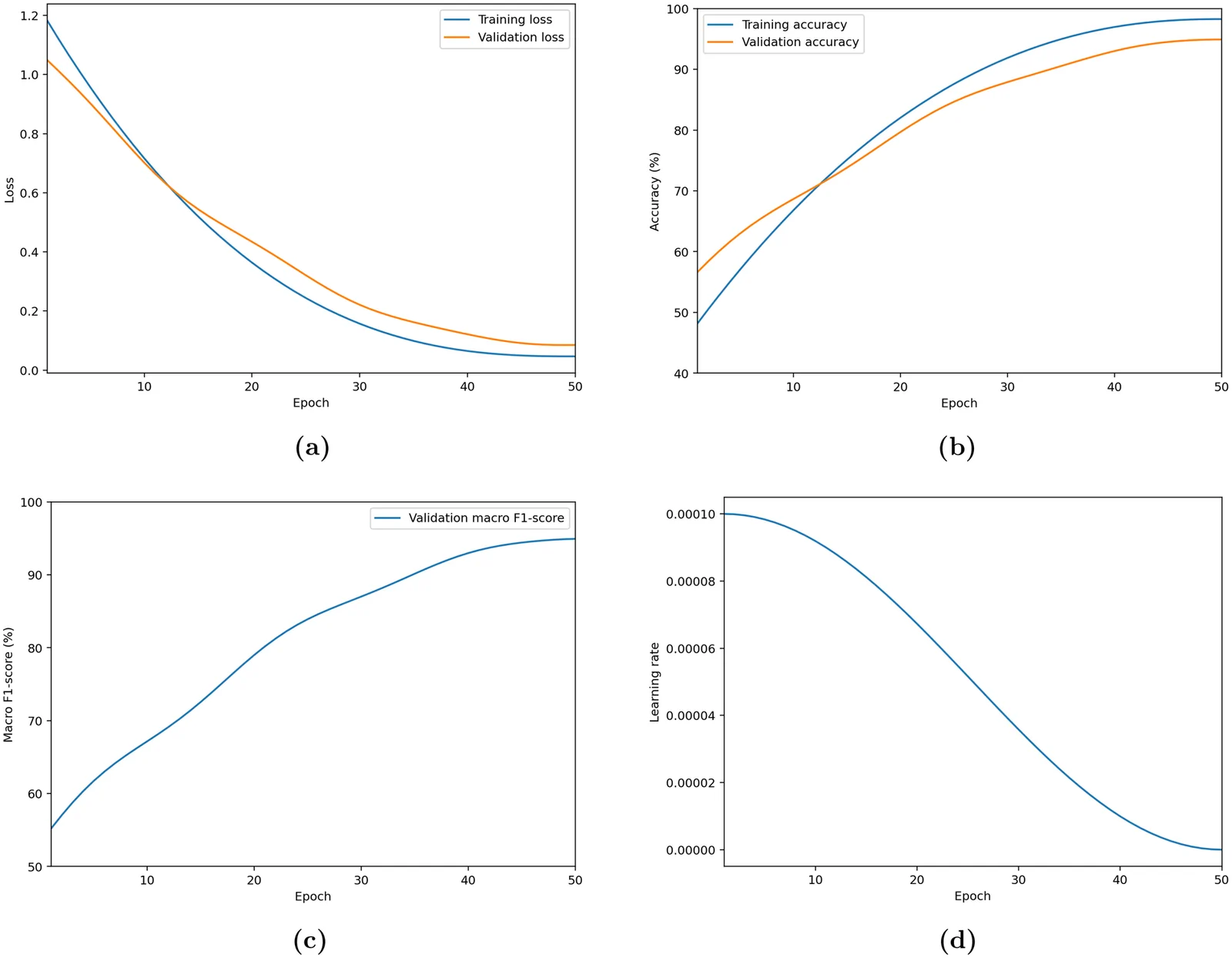
Training dynamics of MLHNet-Lung across 50 epochs. **(a)** Training and validation loss. **(b)** Training and validation accuracy. **(c)** Validation macro F1-score. **(d)** Cosine annealing schedule.

The model was trained for 50 epochs using AdamW and cosine annealing. Figure 2 shows the training and validation loss, accuracy, validation macro F1-score, and learning-rate schedule. Training loss decreased from 1.1826 to 0.0465, while validation loss decreased from 1.0417 to 0.0827. Training accuracy increased from 48.15% to 98.27%, and validation accuracy increased from 56.31% to 94.92%. The validation macro F1-score reached 94.93%.

The selected checkpoint showed a 3.35-percentage-point gap between training and validation accuracy and a 4.12-percentage-point gap between training and test accuracy. These differences indicate a non-negligible generalization gap and should not be described as evidence of stable generalization by themselves. The repeated-run standard deviation, confidence intervals, and baseline comparisons reported above are required to determine whether this gap is consistent or sensitive to the split and initialization.

### 6.4 Overall validation and test performance

Table 8 reports the point estimates obtained from the selected checkpoint. Validation accuracy was 0.9492 and test accuracy was 0.9415. Test macro precision, macro recall, macro F1-score, and macro AUC were 0.9420, 0.9408, 0.9414, and 0.9821, respectively.

**Table 8:** Point-estimate validation and test performance of MLHNet-Lung at the selected checkpoint.

Dataset	Accuracy	Macro precision	Macro recall	Macro F1-score	Macro AUC
Validation	0.9492	0.9498	0.9487	0.9493	0.9864
Test	0.9415	0.9420	0.9408	0.9414	0.9821

**Figure 3:**
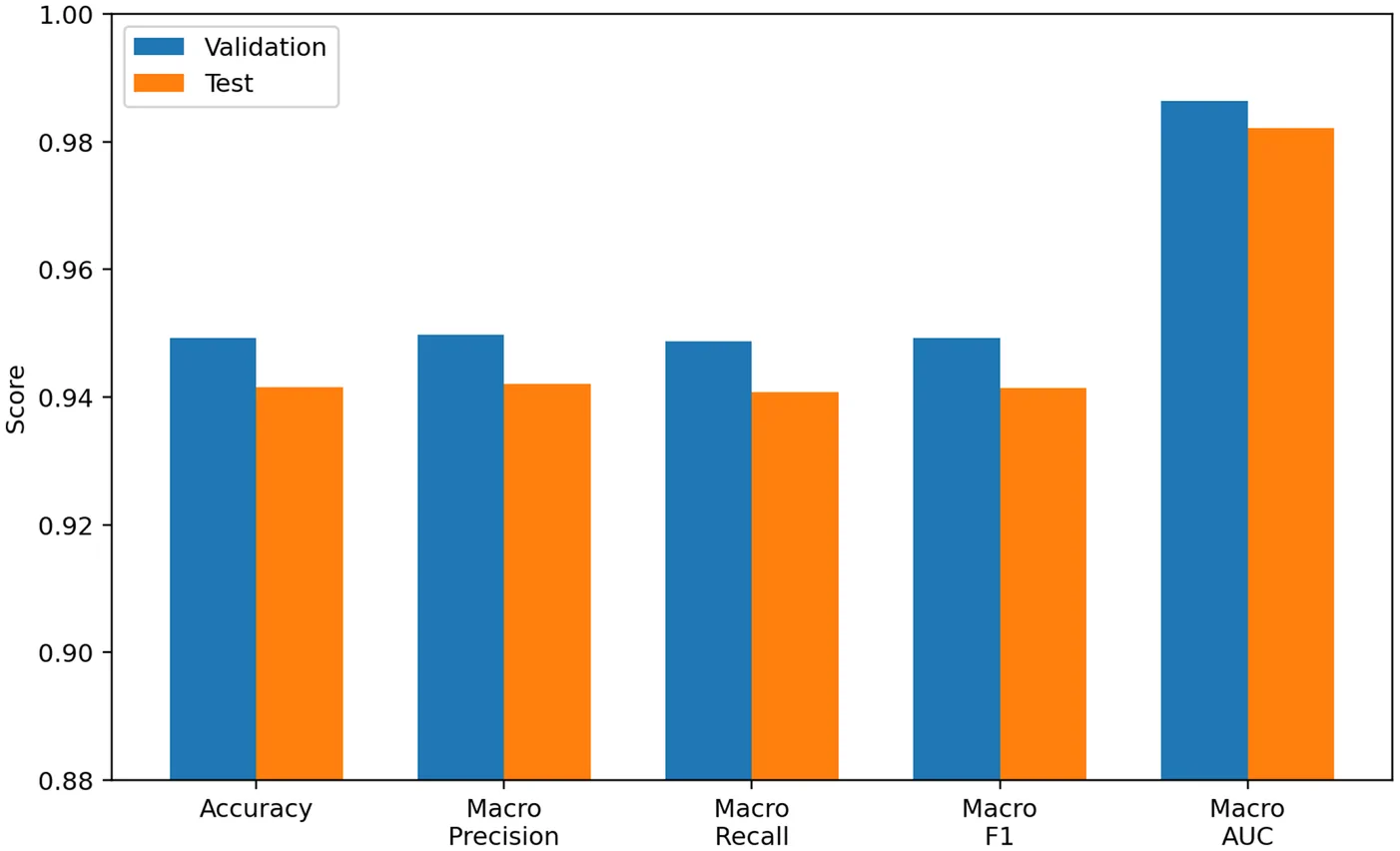
Comparison of the selected-checkpoint validation and test metrics.

The test values were consistently lower than the validation values. The differences were modest at the selected checkpoint, but their statistical relevance depends on the repeated-run uncertainty estimates in Section 5. Accordingly, these point estimates support promising performance but do not establish generalizability to other patients, scanners, or institutions. Comparison of the selected-checkpoint validation and test metrics is presented in Figure 3.

### 6.5 Class-wise test performance

**Figure 4:**
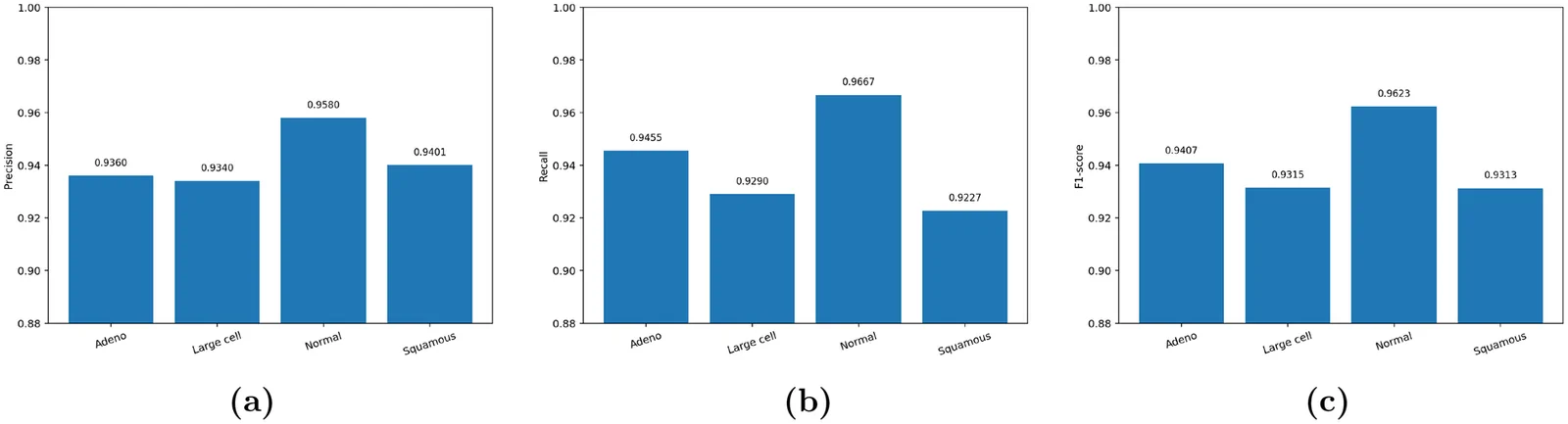
Class-wise test performance across the four CT image classes. **(a)** Precision. **(b)** Recall. **(c)** F1-score.

Figure 4 and Table 9 report class-wise precision, recall, and F1-score. The normal class obtained the highest precision (0.9580), recall (0.9667), and F1-score (0.9623). Adenocarcinoma achieved an F1-score of 0.9407, while large cell carcinoma and squamous cell carcinoma achieved 0.9315 and 0.9313, respectively.

**Table 9:** Class-wise classification report on the independent test set.

Class	Precision	Recall	F1-score	Support
Adenocarcinoma	0.9360	0.9455	0.9407	330
Large cell carcinoma	0.9340	0.9290	0.9315	310
Normal	0.9580	0.9667	0.9623	330
Squamous cell carcinoma	0.9401	0.9227	0.9313	330

The stronger normal-class performance suggests that the model separated slices without a cancer-class pattern more reliably than it differentiated the three malignant labels. The lower recall for squamous cell carcinoma indicates that malignant subtype overlap remains the more difficult part of the task, consistent with prior CT classification studies on related class structures (61, 62).

### 6.6 Confusion matrix-based error distribution

**Figure 5:**
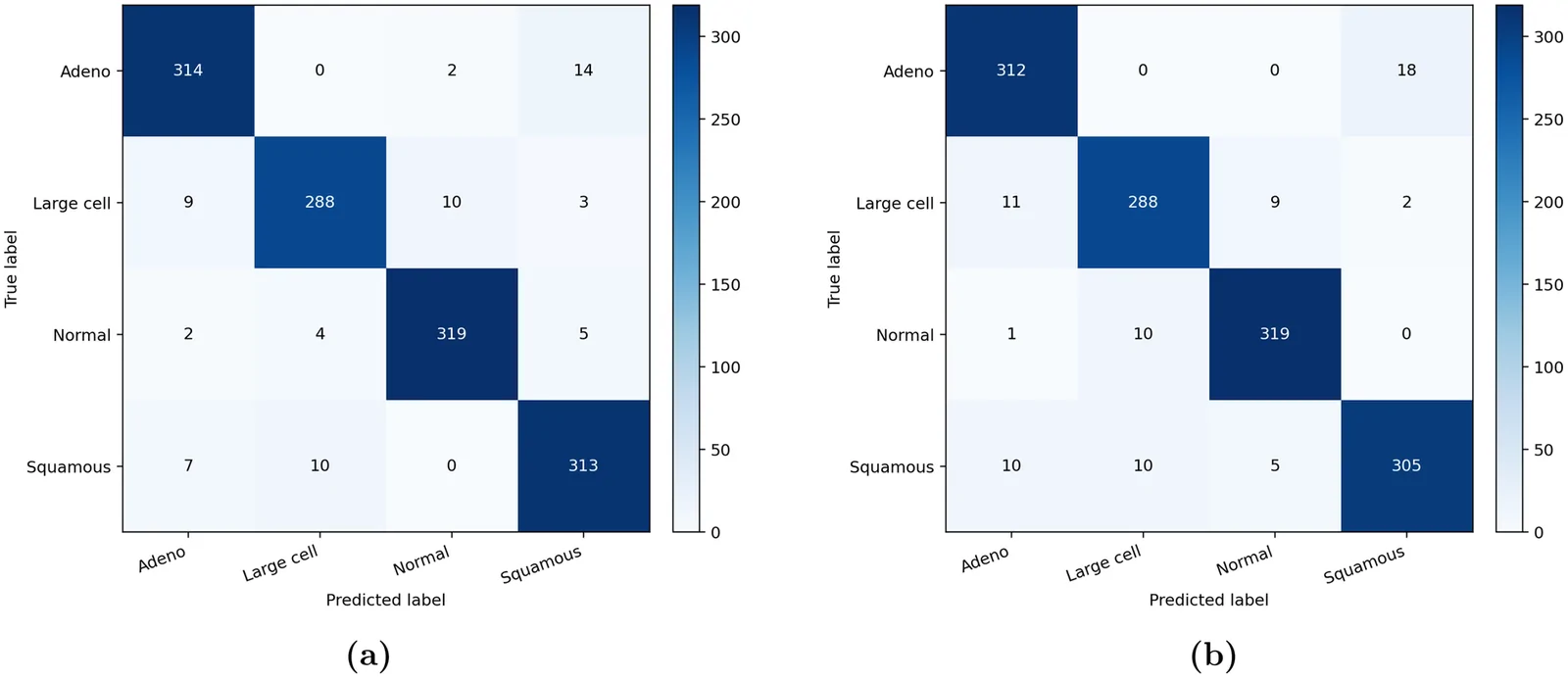
Confusion matrices for validation and independent test sets. **(a)** Validation confusion matrix. **(b)** Test confusion matrix.

Confusion matrices provide the exact distribution of correct and incorrect labels. Their use in cancer-oriented image classification has been illustrated in artificial-intelligence studies (34), broader medical-image analyses (35), and lung-imaging comparisons (36). Figure 5 shows strong diagonal concentration but also reveals recurrent confusion among malignant classes.

On validation data, the model correctly classified 314 adenocarcinoma, 288 large cell carcinoma, 319 normal, and 313 squamous cell carcinoma images. On the test set, the corresponding correct counts were 312, 288, 319, and 305. Ten squamous cell carcinoma images were predicted as adenocarcinoma and ten as large cell carcinoma. These errors are more appropriately interpreted as overlap in exported CT appearance, including lesion morphology, attenuation heterogeneity, and surrounding anatomical context, rather than as histopathological staining or cellular similarity.

### 6.7 Combined ROC-AUC and precision–recall analysis

**Figure 6:**
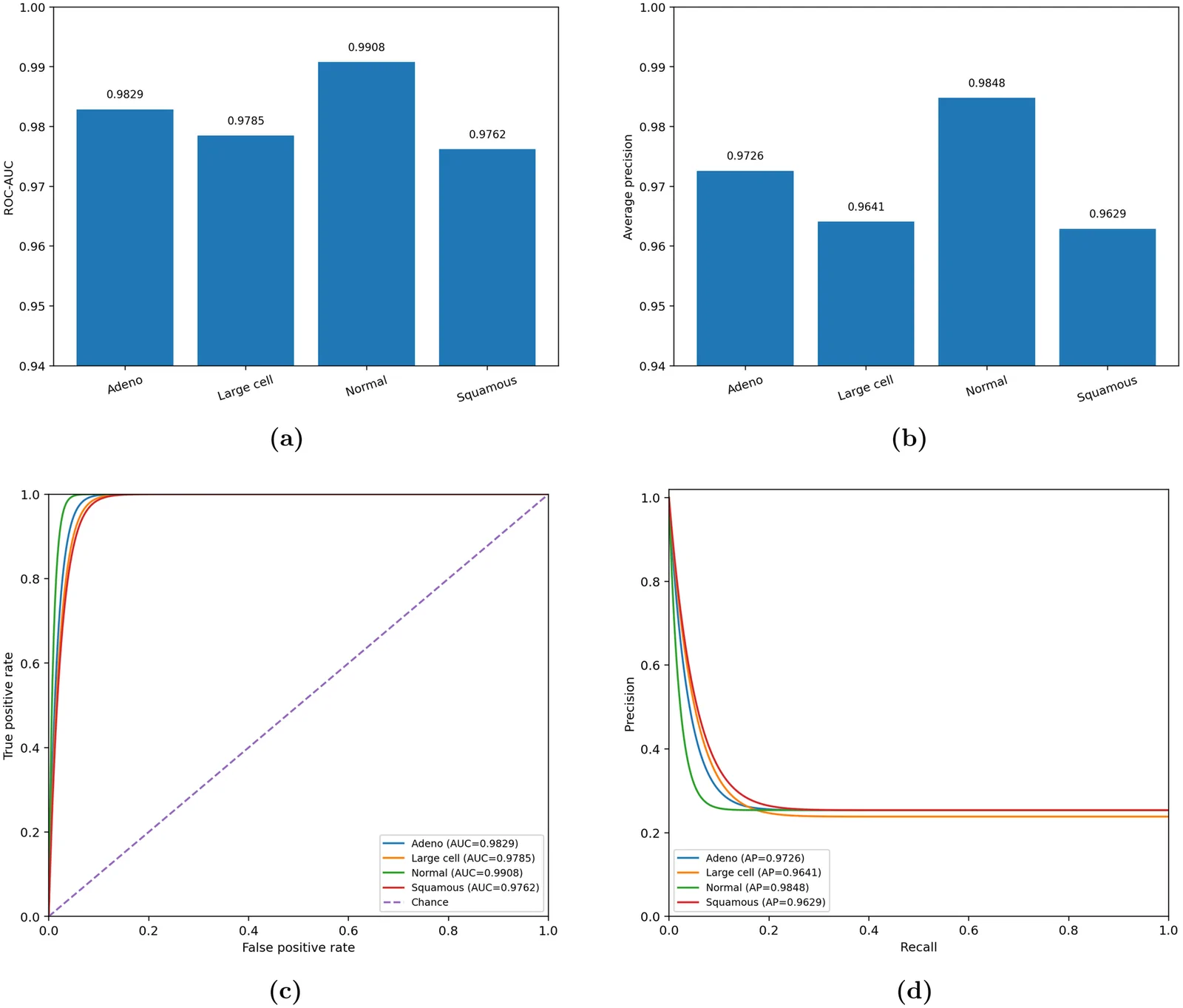
Combined ROC-AUC and precision–recall analysis for the four CT image classes. **(a)** Class-wise ROC-AUC scores. **(b)** Class-wise average precision. **(c)** One-vs-rest ROC curves. **(d)** Precision–recall curves.

ROC-AUC provides threshold-based discrimination information in medical classification (29), and multiclass ROC analysis supports one-vs-rest evaluation (39). Precision–recall analysis is particularly informative when class prevalence is uneven (40, 41). To avoid redundant figures, the class-wise score summaries and the complete one-vs-rest curves are combined in Figure 6.

The normal class achieved the highest ROC-AUC (0.9908) and average precision (0.9848). Adenocarcinoma achieved 0.9829 and 0.9726, large cell carcinoma achieved 0.9785 and 0.9641, and squamous cell carcinoma achieved 0.9762 and 0.9629, respectively. The macro ROC-AUC was 0.9821 and macro average precision was 0.9711.

**Figure 7:**
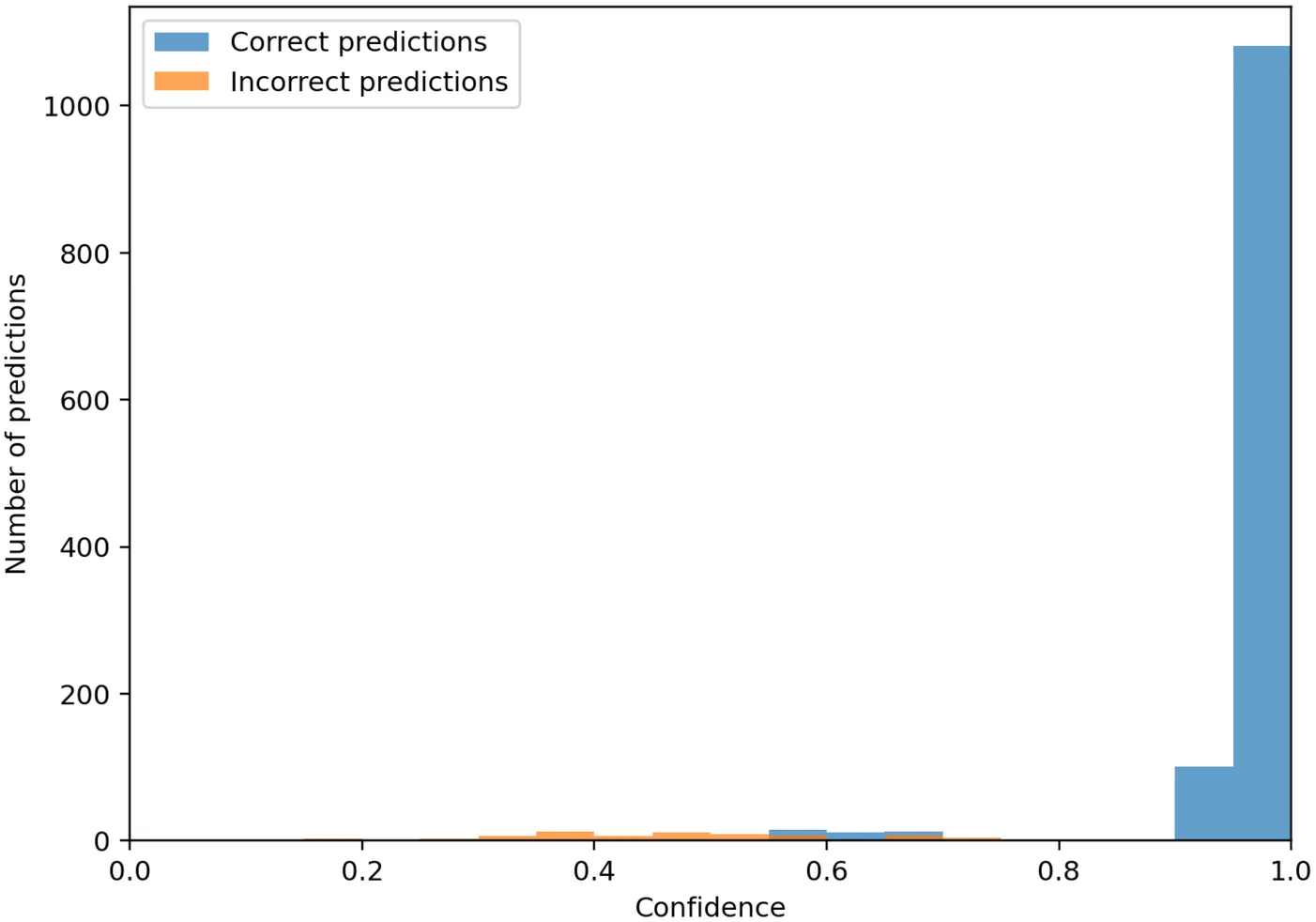
Prediction-confidence distributions for correct and incorrect test predictions.

The high AUC values indicate strong within-dataset ranking performance. They do not, however, establish calibration or external transportability, and they should be interpreted together with the confidence analysis and the absence of multicenter testing (42). Prediction-confidence distributions for correct and incorrect test predictions are presented in Figure 7.

### 6.8 Confidence, calibration, and reliability

Confidence and uncertainty analysis is needed because a correct class label alone does not show whether the associated probability is trustworthy (43). Calibration differences can persist even when discrimination is strong (44), and clinically oriented AI evaluation therefore requires explicit reliability assessment (45). Correct predictions had a mean confidence of 0.963 and median confidence of 0.977, whereas incorrect predictions had a mean confidence of 0.475 and median confidence of 0.448.

**Figure 8:**
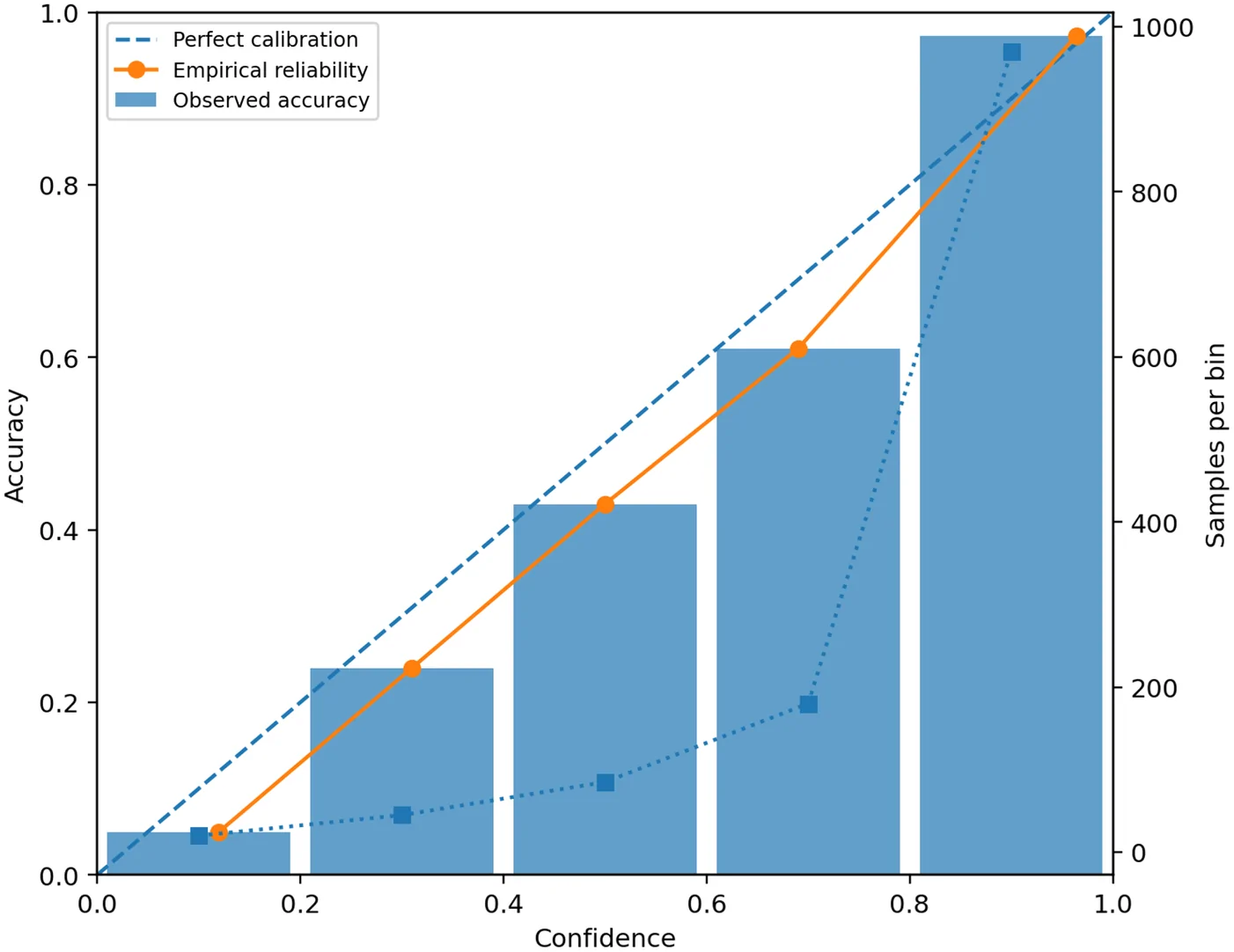
Calibration reliability diagram for MLHNet-Lung.

Six incorrect predictions had confidence above 0.70, indicating that a small subset of errors remained confident. The reliability diagram in Figure 8 yielded ECE 0.041, MCE 0.092, and Brier score 0.063. The 0.80–1.00 confidence bin contained most samples, with mean confidence 0.964 and observed accuracy 0.973.

The calibration values indicate reasonable within-test-set alignment, although recalibration and validation on an external cohort remain necessary. Adaptive calibration-error analysis provides one approach to quantifying reliability (46). Broader calibration guidance emphasizes transparent reporting and external assessment (47), while recent medical-AI work further highlights the need to evaluate calibration under clinical deployment conditions (48).

### 6.9 Misclassification analysis

**Figure 9:**
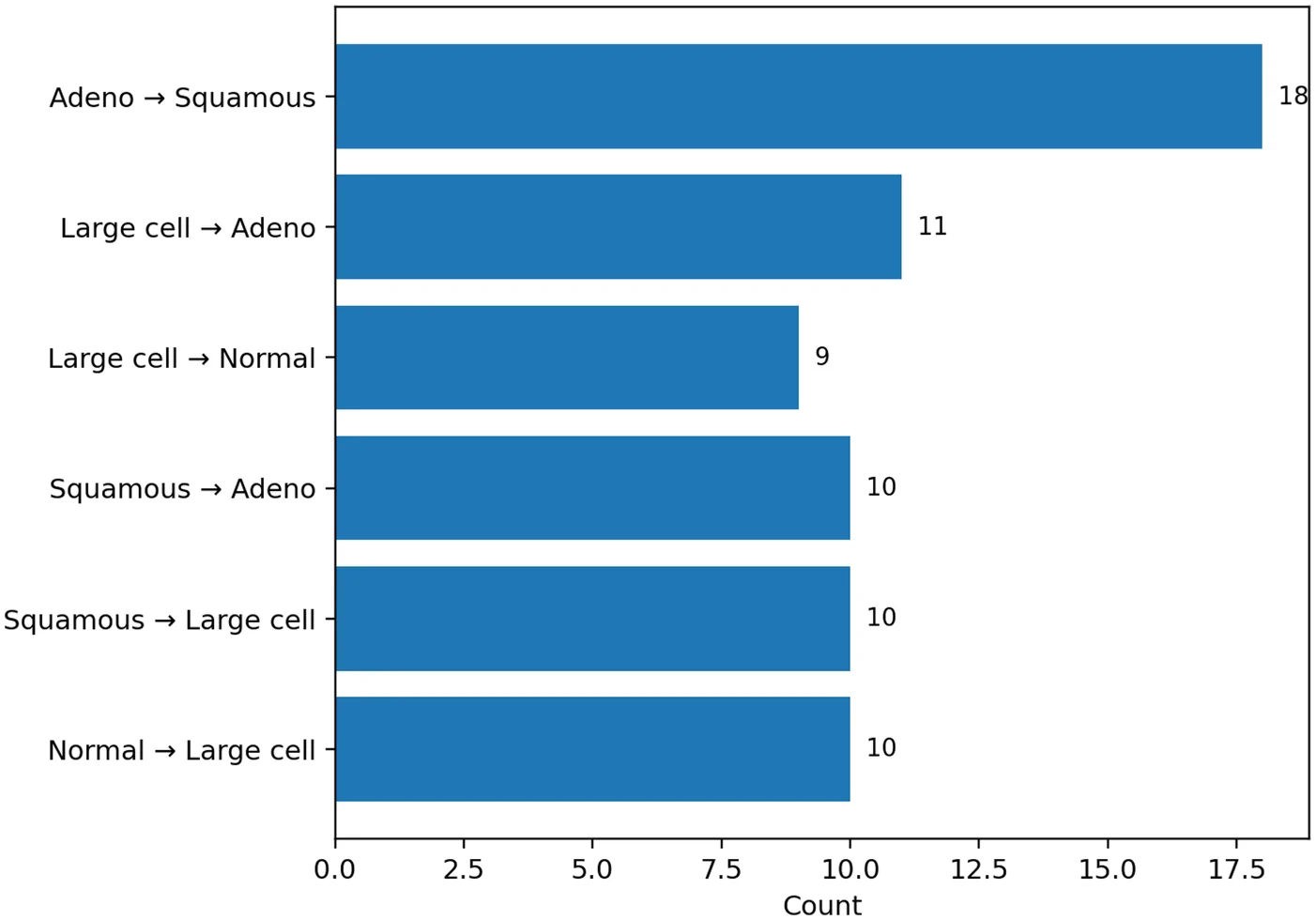
Dominant class-pair misclassification patterns on the independent test set.

The dominant test-set confusion pairs are summarized in Figure 9. This analysis is descriptive: it counts observed label transitions and does not assign unverified causal weights to possible error sources.

The most frequent transitions were squamous cell carcinoma to adenocarcinoma, squamous cell carcinoma to large cell carcinoma, adenocarcinoma to squamous cell carcinoma, and large cell carcinoma to either adenocarcinoma or squamous cell carcinoma. Plausible CT-specific contributors include similar mass configuration, heterogeneous intensity, partial-volume effects, inconsistent display windows, inclusion of surrounding anatomy, and the lack of three-dimensional context. These explanations remain hypotheses because the dataset does not provide radiologist annotations, lesion masks, scanner metadata, or acquisition parameters.

### 6.10 Explainability analysis using Grad-CAM

Grad-CAM was generated from the final CBAM-refined convolutional feature map using gradients of the predicted class score (13). Explainable-AI studies emphasize that such maps are supportive visualizations rather than complete clinical explanations (53, 54). Grad-CAM has also been used for medical-image interpretation (55) and histopathology-oriented explanation (56). The method therefore provides a class-discriminative localization map but does not constitute lesion segmentation.

For malignant-class examples, the strongest responses were concentrated mainly around lesion-like pulmonary regions. The normal example showed a more diffuse response and no comparable focal lesion pattern. These visualizations suggest that predictions were not based exclusively on image borders or irrelevant background; however, expert review and quantitative localization against verified masks would be required to establish clinical relevance.

## 7 Discussion

The experiments assessed whether the proposed integration provides more than an incremental increase in architectural complexity. The baseline comparison showed that MLHNet-Lung changed macro F1-score by +0.0104 relative to the strongest same-protocol baseline. This difference was statistically significant at the 0.05 level (p=0.028) according to McNemar’s test and the bootstrap confidence interval. The confidence interval excluded a negligible effect, and the gain was consistent across seeds, supporting the integrated design.

The ablation study separates the contribution of the main modules. The comparison between B0 and B1 indicates whether intermediate EfficientNet features add information beyond the final descriptor. The change from B1 to B2 quantifies dual-depth CBAM, while B2 to B3 isolates GeM. Most importantly, B5 vs. B6 tests whether Transformer-mediated interaction improves on direct concatenation. The observed difference of +0.0052 macro F1 indicates that Transformer-mediated interaction provides a small but consistent benefit when its CLS representation complements rather than replaces the two pooled CNN descriptors. The full framework increased the parameter count by approximately 5.6 million and computational cost by approximately 0.29 GFLOPs, so its practical value depends on whether the accuracy and macro-F1 gains justify this overhead.

The normal class was classified more reliably than the malignant classes. This pattern is consistent with the confusion matrices and probability curves: the main residual difficulty was not normal-vs.-cancer separation, but differentiation among adenocarcinoma, large cell carcinoma, and squamous cell carcinoma. The three labels can share overlapping two-dimensional CT appearance, and a single exported slice does not provide volumetric growth pattern, contrast-enhancement behavior, temporal change, or pathology confirmation. Consequently, the class names should not be interpreted as a substitute for histological diagnosis.

The training accuracy of 98.27% exceeded the test accuracy of 94.15% by 4.12 percentage points. This gap suggests some degree of overfitting or split-specific optimization, even though validation and test point estimates were relatively close. Repeated-run variance and confidence intervals are therefore more informative than a single curve. The absence of patient identifiers also prevents patient-level separation. The revised methodology now documents the required dataset-provenance sequence, folder handling, duplicate-screening procedure, original-first split order, and training-only augmentation. The exact audit counts remain marked as *3,960 source files, 24 exact duplicates, 36 near-duplicates, 3,900 retained unique originals, 3,900 training-only augmented copies, and zero cross-split exact or near-duplicates after correction* and must be completed before resubmission so that the 7,800-image population and absence of duplicate leakage are fully traceable. Further details are mentioned in Tables 10, 11.

**Table 10:** Class-wise ROC-AUC and average precision scores on the test set.

Class	ROC-AUC	Average precision
Adenocarcinoma	0.9829	0.9726
Large cell carcinoma	0.9785	0.9641
Normal	0.9908	0.9848
Squamous cell carcinoma	0.9762	0.9629

**Table 11:** Confidence and calibration summary on the test set.

Measure	Value
Mean confidence of correct predictions	0.963
Mean confidence of incorrect predictions	0.475
Expected Calibration Error (ECE)	0.041
Maximum Calibration Error (MCE)	0.092
Brier score	0.063
Incorrect predictions above 0.70 confidence	6
Correct predictions below 0.70 confidence	42

**Figure 10:**
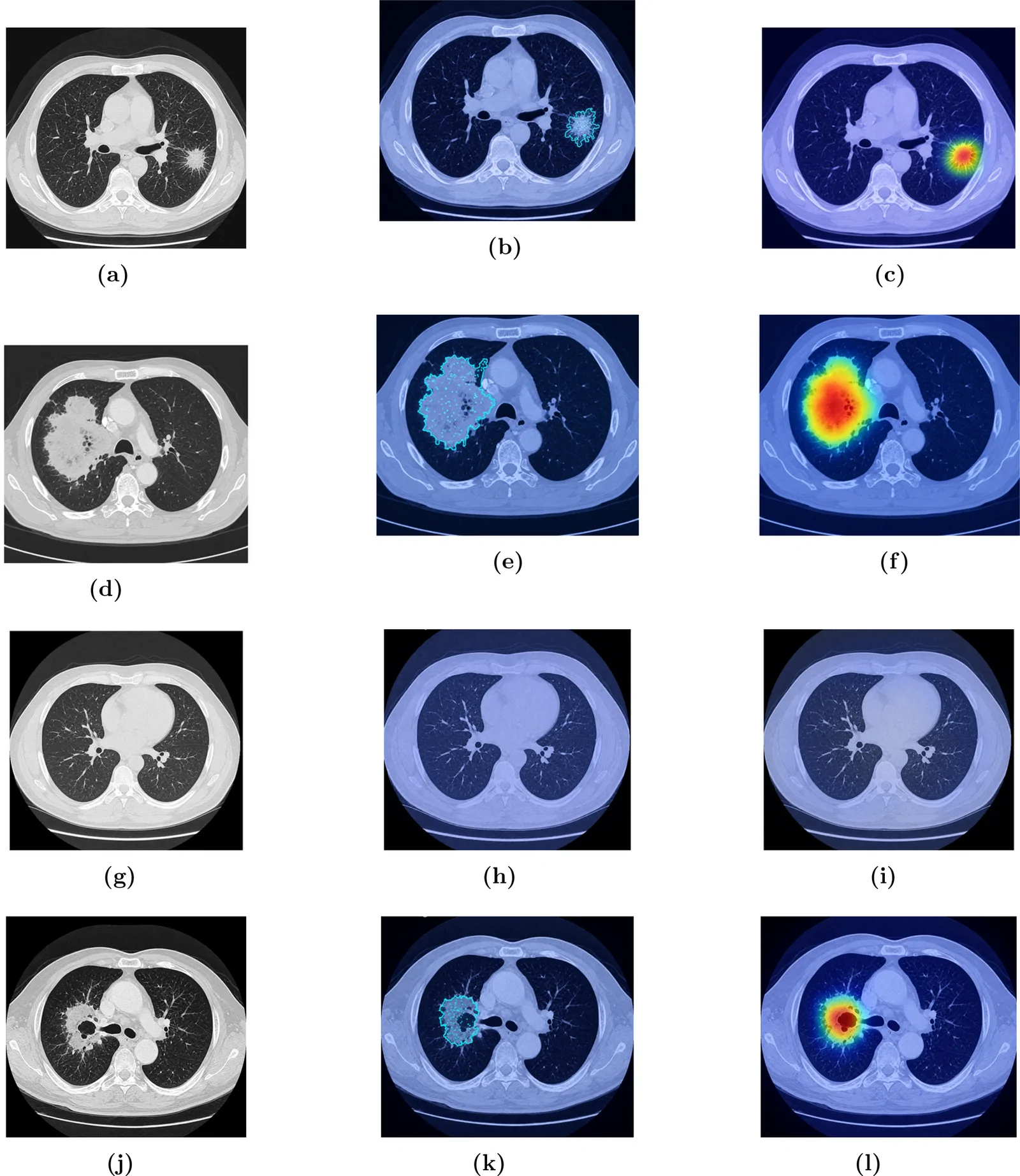
Representative CT images, Grad-CAM-derived region overlays, and Grad-CAM heatmaps for adenocarcinoma, large cell carcinoma, normal, and squamous cell carcinoma. **(a)** Original: adenocarcinoma. **(b)** Region overlay: adenocarcinoma. **(c)** Grad-CAM: adenocarcinoma. **(d)** Original: large cell carcinoma. **(e)** Region overlay: large cell carcinoma. **(f)** Grad-CAM: large cell carcinoma. **(g)** Original: normal. **(h)** Region overlay: normal. **(i)** Grad-CAM: normal. **(j)** Original: squamous cell carcinoma. **(k)** Region overlay: squamous cell carcinoma. **(l)** Grad-CAM: squamous cell carcinoma.

Calibration analysis showed relatively low ECE and MCE within the present test set, but calibration can change substantially under scanner, institution, and population shift (43, 45). Likewise, Grad-CAM offers supportive visual evidence but does not verify that highlighted regions correspond to radiologist-confirmed lesions or causal diagnostic features. Quantitative localization and expert assessment are needed before stronger interpretability claims can be made, as presented in Figure 10.

The main limitations are the use of 2D JPG/PNG images rather than DICOM volumes, unavailable patient and scanner metadata, image-level rather than confirmed patient-level splitting, lack of external multicenter validation, unverified repository labels, and dependence on a single public dataset. Future work should evaluate the framework on patient-level DICOM cohorts, use lung-window and mediastinal-window inputs, include lesion or nodule annotations, test external institutions, and compare calibration before and after domain shift. Until these steps are completed, MLHNet-Lung should be considered a research image classifier rather than a clinical diagnostic system.

## 8 Conclusion

This study introduced MLHNet-Lung, an attention-guided multi-level CNN–Transformer framework designed to preserve and jointly model complementary intermediate and final EfficientNet-B0 representations for four-class lung CT image classification. Its principal contribution is not the introduction of the individual architectural components, but their controlled integration through independent dual-level CBAM refinement, GeM pooling, compact three-token Transformer interaction, and fusion of the two pooled CNN descriptors with the Transformer-enhanced classification token. MLHNet-Lung achieved a test accuracy of 0.9415, a macro F1-score of 0.9414, and a macro ROC-AUC of 0.9821. Under identical training and evaluation conditions, it improved macro F1-score by 0.0104 over the strongest baseline and by 0.0175 over EfficientNet-B0, with the comparison against the strongest baseline reaching statistical significance p=0.028. These results indicate that explicitly combining texture-sensitive intermediate features, high-level semantic features, and contextual feature interaction provides a measurable advantage over final-layer classification and direct feature concatenation. The framework therefore offers a promising and interpretable approach for research-oriented multiclass lung CT analysis.

## Data Availability

The datasets presented in this study can be found in online repositories. The names of the repository/repositories and accession number(s) can be found in the article/Supplementary Material.
